# Phenotypic screening, transcriptional profiling, and comparative genomic analysis of an invasive and non-invasive strain of *Candida albicans*

**DOI:** 10.1186/1471-2180-8-187

**Published:** 2008-10-24

**Authors:** Sascha Thewes, Gary P Moran, Beatrice B Magee, Martin Schaller, Derek J Sullivan, Bernhard Hube

**Affiliations:** 1 Division FG16 Mycology, Robert Koch Institute, Berlin, Germany; 2 Microbiology Research Unit, Division of Oral Biosciences, Dublin Dental School & Hospital, Trinity College Dublin, University of Dublin, Dublin 2, Republic of Ireland; 3 Department of Genetics, Cell Biology, and Development, University of Minnesota, Minneapolis, MN, USA; 4 Department of Dermatology, University of Tübingen, Tübingen, Germany; 5 Department of Microbial Pathogenicity Mechanisms, Leibniz Institute for Natural Product Research and Infection Biology – Hans Knoell Institute, Jena, Germany; 6 Friedrich-Schiller-University, Jena, Germany; 7 Present address: Institute for Biology – Microbiology, Department of Biology, Chemistry, Pharmacy, Freie Universität Berlin, Berlin, Germany

## Abstract

**Background:**

Invasion of host tissue by the human fungal pathogen *Candida albicans *is an important step during the development of candidosis. However, not all *C. albicans *strains possess the same invasive and virulence properties. For example, the two clinical isolates SC5314 and ATCC10231 differ in their ability to invade host tissue and cause experimental infections. Strain SC5314 is invasive whereas strain ATCC10231 is non-invasive and strongly attenuated in virulence compared to SC5314. In this study we compare the *in vitro *phenotypic, transcriptional and genomic profiles of these two widely used laboratory strains in order to determine the principal biological and genetic properties responsible for their differential virulence.

**Results:**

In all media tested, the two strains showed the same metabolic flexibility, stress resistance, adhesion properties and hydrolytic enzyme secretion *in vitro*. However, differences were observed in response to cell-surface disturbing agents and alkaline pH. Furthermore, reduced hyphal formation in strain ATCC10231 under certain conditions correlated with reduced invasive properties in an *in vitro *invasion assay and a reduced ability to invade epithelial tissue. Despite these diverse phenotypic properties, no substantial genomic differences were detected by comparative genome hybridisation within the open reading frames. However, *in vitro *transcriptional profiling displayed major differences in the gene expression of these two strains, even under normal *in vitro *growth conditions.

**Conclusion:**

Our data suggest that the reason for differential virulence of *C. albicans *strains is not due to the absence of specific genes, but rather due to differences in the expression, function or activity of common genes.

## Background

*Candida albicans *is a commensal of the normal human microflora but can also cause a variety of infections ranging from superficial mucosal infections to haematogenously disseminated infections. In order to reach the bloodstream, *C. albicans *has to cross physical barriers such as epithelial cell layers by active penetration and/or induced endocytosis [1]. Once in the bloodstream the fungus may disseminate throughout the entire body often causing systemic multi-organ infections [2].

During the different stages of a *C. albicans *infection and within different host tissues and environments, the fungus has to express general as well as stage- and tissue-specific virulence or fitness factors (reviewed in [3]). The first step for successful colonisation of mucosal surfaces or any other tissue by *C. albicans *is adhesion. Some factors involved in adhesion also have additional roles in tissue invasion by *C. albicans*. For example the glycosylphosphatidylinositol (GPI) – protein Als3 mediates attachment to endothelial cells [4], but binding of this protein to host cell cadherins also leads to invasion via induced endocytosis of the fungal cells [5]. Other adhesion proteins such as Hwp1 are important for the establishment of a tight, covalent connection between the fungus and the host [6,7]. In contrast to pathogenic bacteria, *C. albicans *not only induces its own endocytosis, but also actively penetrates through host tissues. Secreted hydrolytic enzymes may aid the fungus during invasion by destroying host cell surface components and immune factors or by extracting nutrients for the fungus [8]. Among these hydrolases are secreted aspartic proteases (Saps), phospholipases (Plbs) and lipases (Lips) [9]. Another important virulence attribute of *C. albicans *is the ability to switch between ovoid yeast and filamentous hyphal growth forms. It has been proposed that yeast cells are more suitable for dissemination whereas hyphal forms play a key role during invasion [10]. Supporting this view, most mutants which have reduced abilities to undergo the yeast to hyphal transition are attenuated in a wide range of infection models [1,11-13]. Other proteins that have been implicated in the pathogenesis of *C. albicans *infections are associated with metabolic activity. The acquisition of nutrients plays a central role during infection and therefore it is not surprising that proteins that are involved in glucose and nitrogen sensing also contribute to fungal fitness and virulence [14,15]. Furthermore, the availability of free iron is essential for *C. albicans *and deletion of the gene *FTR1*, which encodes a high affinity iron permease, leads to avirulence of this mutant in a murine model of systemic infection [16]. Additionally, the ability to sense and adapt to changing environments such as alkaline or acidic milieus are important virulence properties of *C. albicans*. For example, it has been shown that the correct sensing of extracellular pH is a prerequisite for hyphal formation and virulence under alkaline conditions and during systemic infections [13,17,18].

Despite the clonal mode of reproduction of *C. albicans*, there are clear differences between clinical isolates and not all strains of *C. albicans *have the same biological properties. *C. albicans *strain typing protocols have been developed to provide information on the sources, carriage, and transmission of infection, in addition to information on the relationship between genotype and phenotypic properties such as virulence and antimicrobial resistance [19]. More recently, traditional strain typing based on microbial phenotypes [20-22] has been combined with different genotypic typing methods. Multilocus sequence typing (MLST) was used to show that adult patients with bloodstream infections are usually infected with their own endogenous commensal isolates [19] and to provide evidence that clades are associated with geographical sources [23]. Whether certain clades or natural genetic variation in *C. albicans *strains can be associated with different virulence capabilities remains a matter of debate.

In addition to large fingerprinting studies of several hundred *C. albicans *strains, other studies have analysed specific *C. albicans *strains in more detail. The best characterised strain is *C. albicans *SC5314 [24]. This clinical isolate recovered from a patient in the 1970s was the first strain which was used for targeted genetic manipulations [25] and because it is one of the most commonly used laboratory strains it was also the first strain chosen for complete genome sequencing [26]. Other *C. albicans *strains have been successfully used as reference strains in biochemical or pharmacological studies. One of these strains, *C. albicans *ATCC10231, is a clinical strain isolated during the 1960s and was used in early infection model studies [27,28]. Strain ATCC10231 is still frequently used for pharmacological purposes [29-31] although several studies have shown that this strain produces shorter germ tubes compared to strain SC5314, is non-invasive in an intraperitoneal infection model and in an *ex vivo *infection model [32-34], and is strongly attenuated in all other animal models tested [27,28,35]. Based on MLST it was shown that strain SC5314 belongs to clade 1 whereas strain ATCC10231 belongs to clade 2 [23]. However, both strains also share common genetic features. For example, both strains are heterozygous at the *MTL*-locus and both strains belong to genotype A on the basis of rRNA analysis [23]. Although significant progress has been made in the population genetics of clinical *C. albicans *isolates, it is not understood why there are such apparent differences between strains such as ATCC10231 and SC5314 in their capacity to cause infection.

One possible way of identifying genes and factors associated with the ability of microbes to cause infection is to investigate closely related strains or species which differ in their potential to cause disease. Such strategies have been successfully used for the characterisation of pathogenic and non-pathogenic amoebae (reviewed in [36]), for the investigation of slow and fast growing mycobacteria (reviewed in [37]) and for other pathogenic and non/less-pathogenic bacteria [38,39]. Recently comparative genomic hybridisation (CGH) was used to compare the genes present, absent or divergent in the genomes of the closely related species *C. albicans *and *C. dubliniensis*, the latter of which is rarely found in clinical samples [40].

In this study, we used phenotypic screening, transcriptional profiling and comparative genomics to compare the invasive and highly virulent *C. albicans *strain SC5314 with the non-invasive and comparatively less virulent strain ATCC10231 to gain a better insight into principal biological and genetic properties responsible for the differential virulence of these *C. albicans *strains under defined *in vitro *conditions. We have identified minor as well as major differences between the strains at the phenotypic level under various conditions. These differences are not due to loss of genes in the less virulent strain. Although sequence variations between homologous genes in each strain exist, these are infrequent and limited in their extent. Nevertheless, we found substantial differences in the *in vitro *transcriptional profiles of the two strains under even normal growth conditions.

## Methods

### Strains, media and phenotypic screens

*C. albicans *strains SC5314 [24] and ATCC10231 [32] and *S. cerevisiae *strain JS91.15-23 [41] were routinely grown on YPD plates (1% (w/v) peptone, 1% (w/v) yeast extract, 2% (w/v) glucose, 2% (w/v) agar).

For phenotypic screens, strains were incubated on SD plates (0.17% (w/v) yeast nitrogen base (BD Bioscience), 0.5% (w/v) ammonium sulfate, 2% (w/v) glucose, 2% (w/v) agar) supplemented with the indicated substances (Table 1) and incubated at 30°C and 37°C for up to five days. YP medium without glucose was prepared as described previously [42]. Invasion into agar surfaces was determined by scraping off non-invasive cells followed by washing of the agar surface with water.

**Table 1:** Growth conditions used for phenotypic characterisation of *C. albicans *strains SC5314 and ATCC10231

**Condition tested**	**Medium/Supplementation**^a^
Control	SD and YPD without any supplementation
Carbon source^b^	galactose, glycerol, mannitol, **ethanol**^c ^(2% (w/v) each)
pH value	pH 4, pH 5, pH 6, pH 7, pH 8
Temperature	18°C, 30°C, 37°C, 42°C
Elevated cation concentration	NaCl (1 M and 1.3 M), CaCl_2 _(50 mM and 300 mM)
Anaerobic growth	anaerobic jar, embedded conditions (YPS [78])
Hyphae induction	10% foetal calf serum, Lee's medium [79], Spider medium [80], GlcNac, embedded (YPS), SLAD [81]
Resitance towards antimycotics	hygromycin B (400 μg/ml), amorolfin (3 μg/ml), **itraconazole **(1 μg/ml), caspofungin (200 ng/ml)
Stress	calcofluor white (800 μg/ml), benomyl (200 μg/ml), congo red (100 μg/ml and 150 μg/ml), cyclosporin A (5 mM and 10 mM), NaF (30 mM and 40 mM), **5-FOA **(0.05% (w/v)), caffeine (20 mM), SDS (0.01% (w/v))
Extracellular enzyme activity	BSA agar, egg yolk agar

^a^If not otherwise indicated, the basic medium was SD and media were incubated at 30°C and 37°C.

^b^To test the ability for utilizing different carbon sources, the glucose in the SD medium was exchanged with the same amount of the indicated carbon source.

^c^Supplements in bold are those that showed significant different growth of the two strains.

YPD media buffered at pH 5 or pH 8 and depleted for iron were prepared as described containing 150 mM bathophenanthroline disulphonate (BPS) [43]. Cation sensitivity was tested using YPD medium buffered with 100 mM HEPES at pH 5 or pH 8 and supplemented with 1 M NaCl. Control plates were buffered with 100 mM HEPES at pH 5 and pH 8 without supplementation. Hyphal formation was investigated on M199 (Sigma) plates buffered at pH 8 and N-acetylglucosamine (GlcNac) plates [44]. SD medium depleted for inorganic phosphate (P_i_-depleted) was prepared as described [45] and the pH value adjusted to pH 5 or pH 8.

For the determination of growth rates, *C. albicans *strains were grown over night at 30°C in liquid SD and YPD medium, respectively. For main cultures, fresh medium (SD or YPD) was inoculated with cells from the pre-culture to an OD_600 _of 0.05. Growth rate was determined at 37°C and 180 rpm by measuring OD_600 _at the indicated time points (n = 4 independent experiments).

### Adhesion assay

Epithelial cells (HEp-2) and fibroblasts (NIH/3T3) were used for adhesion assays. HEp-2 cells were routinely grown in RPMI-1640 (Biochrom) supplemented with 10% foetal calf serum (FCS) at 37°C and 5% CO_2_. NIH/3T3 cells were grown in Dulbecco's modified Eagle medium (DMEM; Biochrom) supplemented with 15% FCS at 37°C and 5% CO_2_.

For adhesion assays 1 × 10^5 ^HEp-2 and 3T3 cells, respectively, were seeded in 1 ml medium into 24-well plates and incubated over night at 37°C and 5% CO_2_. The medium was aspirated, cell layers were washed with PBS and overlaid with fresh medium without FCS. After inoculation with 5 μl (5 × 10^5 ^cells) *Candida *cells from a mid-logarithmic YPD-culture, plates were centrifuged for 1 min at 800 g to facilitate cell-cell contact. After 1 h incubation at 37°C and 5% CO_2 _the supernatant was aspirated and non-adherent *Candida *cells were washed away with PBS. Adherent cells were released from the monolayer by adding 200 μl of 0.1% Triton X-100, diluted and spread on YPD plates. After over night incubation at 37°C colony forming units (cfu) were counted.

### Infection of reconstituted human oral epithelium and blood survival assay

Infection of reconstituted human oral epithelium (RHE) was done as described [46]. Briefly, RHE (0.5 cm^2^; Skinethics) was overlayed with 50 μl *Candida*-suspension and incubated up to 48 h at 37°C and 5% CO_2_. At the indicated time points the maintenance medium was aspirated and stored at -20°C until LDH-activity was determined [46]. Infected epithelia were cut out and fixed in 2.5% glutaraldehyde containing 2% (v/v) para-formaldehyde in PBS. Embedding and staining was performed as described previously [47].

To analyse the survival of *C. albicans *cells in whole human blood we used the protocol recently described by Fradin *et al. *[48,49].

### Genomic DNA extraction and labelling

High-molecular-mass total genomic DNA was recovered from *C. albicans *strains by organic extraction following digestion of the cell wall with Zymolyase 20T (Seikagaku) and proteinase K treatment (Roche Diagnostics) as described previously [50].

Labelling of genomic DNA was performed as described [40]. Briefly, 3 μg DNA of each *C. albicans *strain were fragmented by restriction endonuclease digestion with *Tru*1l and *Rsa*I (1.5 μg DNA for each restriction). These digests were then heat inactivated, extracted once with a mixture of phenol/chloroform/isoamyl alcohol (25:24:1) and ethanol precipitated. The two digests were combined and a labelling reaction was carried out using the RadPrime DNA Labelling Kit (Invitrogen) incorporating Cy3 dUTP or Cy5 dUTP. After labelling, reaction products were purified and concentrated.

### Comparative genome hybridisation and sequencing

Labelled DNA-fragments from strain SC5314 and strain ATCC10231 were combined (final volume 25 μl), denatured for 5 min at 95°C and hybridisation and washing of *C. albicans *microarrays (Eurogentec) was carried out as described previously [40]. Each strain was hybridised in duplicate including one dye swap.

Immediately after washing, slides were scanned with an Axon 4000B scanner and data were extracted using GenePix 4.1 (Axon). Raw data have been deposited in NCBIs Gene Expression Omnibus (GEO, ) and are accessible through GEO Series accession number GSE10690. Data were transferred into GeneSpring 7.2 (Agilent) and normalised using LOWESS. Genes were called "absent/divergent" in strain ATCC10231 if the normalised signal intensity was at least 2-fold lower compared to the intensity of strain SC5314. "Absent/divergent" genes were PCR amplified from strain ATCC10231 (primer list see additional file 1) and sequenced using the ABI BigDye 3.1 chemistry (Applied Biosystems) on a 3100 Avant Genetic Analyzer (ABI PRISM, Applied Biosystems). Additionally, five genes that gave a stronger signal on the microarrays with DNA from strain ATCC10231 compared to strain SC5314 were included in the analysis. Sequences were analysed using Chromas 1.45 (Technelysium Pty Ltd) and the Lasergene 6 software package (DNASTAR Inc).

### RNA extraction and labelling

For transcriptional profiling of strains SC5314 and ATCC10231, both strains were grown to OD_600 _of 0.15 in SD medium at 37°C. Cells were centrifuged and the resulting pellet was immediately frozen in liquid nitrogen and stored at -80°C until further use. Total RNA was extracted as described [49] and stored for either cDNA labelling or northern blotting at -80°C.

Labelled cDNA for transcriptional profiling was produced by incubating 30 μg (22 μl) total RNA of each strain with 1 μg oligo-d(T)-primer (Invitrogen) at 70°C for 10 min. After adding 30 μl of labelling master mix (1× reverse transcriptase buffer, 1 mM dithiotreithol, 500 μM dATP, 500 μM dCTP, 500 μM dGTP, 100 μM dTTP; Invitrogen), 3 μl Cy3-dUTP (Amersham) or 3 μl Cy5-dUTP (Amersham), respectively, and 40 U RNaseOut™ (Invitrogen), labelling occurred after addition of 200 U SuperScript™ II reverse transcriptase (Invitrogen) for 2 h at 42°C. After 1 h of incubation 200 U of fresh reverse transcriptase were added. The reaction was stopped by adding 3 μl EDTA (20 μM) and RNA was degraded by addition of 3 μl NaOH (500 mM) following an incubation at 70°C for 10 min. The reaction was neutralised by adding 3 μl HCl (500 mM). Labelled cDNA was washed and concentrated and stored at -20°C until further use.

### Transcriptional profiling

For transcriptional profiling *C. albicans *microarrays (Eurogentec) were prehybridised for 45 min at 42°C in 5 × SSC, 1% (w/v) SDS and 1% (w/v) BSA. Prehybridised microarrays were washed 5× with water, once with isopropanol and the washed arrays were air-dried. Before hybridisation, Cy3- and Cy5-labelled cDNA was mixed, made up to a final volume of 25 μl with water and denatured for 5 min at 95°C. Twenty-five microliters of hybridisation buffer (50% (v/v) formamide, 10 × SSC, 0.2% SDS) were added. The mixture was applied to the prehybridised microarrays, covered with a LifterSlip (25 × 44 inch; Erie Scientific Company), and hybridisation took place over night in a humidified chamber (Corning) in a water bath at 42°C. Hybridised microarrays were washed for 15 min in 2 × SSC, 1% (w/v) SDS, for 8 min in 1 × SSC, 0.2% (w/v) SDS, and for 5 min in 0.1 × SSC, 0.2% SDS. Hybridisation for each strain was done in triplicate including one dye swap.

After washing, the microarrays were immediately scanned with an Axon 4000B scanner at a resolution of 10 μm and data were extracted using the GenePix 4.1 software package (Axon). Extracted data were normalised (LOWESS) and analysed in GeneSpring 7.2 (Agilent). Genes were identified as differentially expressed using Student's t-test with *P *≤ 0.05. All raw data are accessible through GEO Series accession number GSE10689. Expression of selected genes was confirmed by northern blot analysis (data not shown).

### Ammonium uptake assay

For the determination of ammonium uptake, both *C. albicans *strains were incubated over night in minimal proline medium (Mpro; 0.1% (w/v) proline, 0.17% (w/v) yeast nitrogen base without amino acids and ammonium sulfate (BD Bioscience), 2% (w/v) glucose) at 37°C. Cultures were diluted in fresh Mpro to an OD_600 _of 1.0 and supplemented with ammonium sulphate to a final concentration of 1 mM. Cultures were incubated at 37°C on a rotary shaker and at the indicated time points aliquots were taken, centrifuged, and the concentration of free ammonium in the supernatant was determined with a L-glutamate dehydrogenase enzyme test (Sigma). Measurements were done in triplicate and the experiment was repeated. Statistical analysis was done using Student's t-test.

## Results

### Phenotypic comparison of SC5314 and ATCC10231

**Figure 1:**
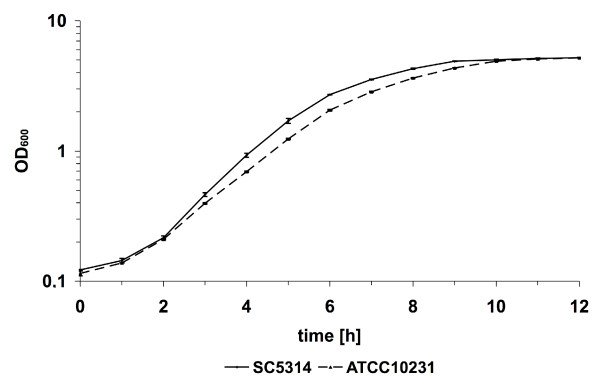
**Growth characteristics of *C. albicans *strains SC5314 and ATCC10231 in SD medium.** Representative figure of n = 4 experiments. Both strains showed similar growth curves in YPD medium (data not shown).

**Figure 2:**
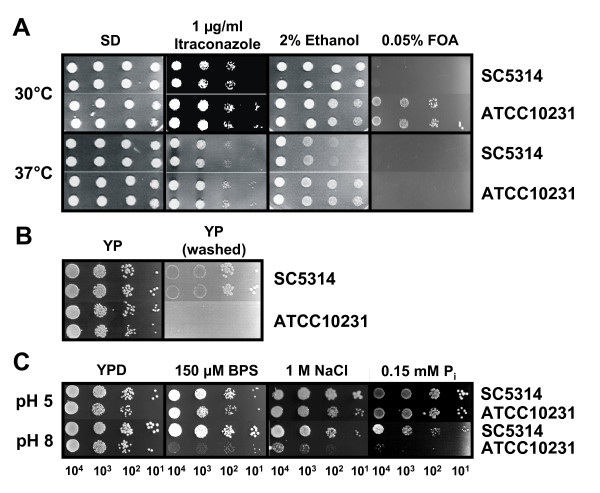
**(A) Growth of *C. albicans *strains SC5314 and ATCC10231 under different stress conditions (addition of itraconazole, ethanol, 5-FOA).** Growth on SD plates served as a control. Both strains were tested at 30°C and at 37°C. **(B) **Invasion of SC5314 and ATCC10231 into carbon-depleted agar surface. After growth of the colonies, plates were washed with water removing the non-invasive cells and photographed. **(C) **Influence of different pH-values on the growth of SC5314 and ATCC10231 at iron- (150 μM BPS) and phosphate-limiting (0.15 mM P_i_) conditions and under cation stress (1 M NaCl). Growth on YPD at pH 5 and pH 8, respectively, served as a control. Numbers under the single colonies represent the number of cells used for inoculation of each spot (also related to A and C).

Characteristics such as growth rate are likely to have an impact on the pathogenicity of different *C. albicans *strains. To elucidate whether the different virulence potential of strains SC5314 and ATCC10231 may be a consequence of differential growth rates, we incubated both strains under a number of growth conditions and monitored their growth. Strains SC5314 and ATCC10231 had slightly different growth rates in defined (SD) and complex (YPD) media at 37°C. Both strains reached similar final cell densities, although strain ATCC10231 remained slightly longer in the lag-phase than strain SC5314 (Fig. 1). Additionally, strain ATCC10231 had a slightly slower generation time during exponential growth (approximately 60 min for strain SC5314 and 72–75 min for strain ATCC10231). This difference in the rate of growth was accounted for in all subsequent investigations by using identical cell numbers obtained from the same growth phase rather than cells from the same time point of culture growth. To test the metabolic flexibility and the response of both strains to environmental stress, the strains were incubated under a variety of different conditions on agar plates. These tests included growth with different carbon sources, at different pH values (pH 4–8), at different temperatures (18°C–42°C), on agar plates supplemented with elevated cation concentrations or antifungal agents, under anaerobic conditions, or under other conditions that caused cellular stress (e.g. cell wall stress; Table 1). Under most of these conditions both strains showed identical growth properties (data not shown). However, under certain conditions tested we observed significant differences in the susceptibility of the two strains towards specific substances. For example, the less virulent strain ATCC10231 was more tolerant to the cell-surface-integrity affecting substances itraconazole and ethanol at 37°C or 5-fluoroorotic acid (5-FOA) at 30°C compared to strain SC5314 (Fig. 2A).

In a previous study, Kretschmar *et al. *showed that strain ATCC10231 produced shorter germ tubes than strain SC5314 in media containing foetal calf serum [32]. However, since germ tube formation can be induced by several factors, we investigated hyphal formation by applying additional protocols known to induce filamentous growth, such as agar plates containing N-acetyl glucosamine (GlcNac) and M199-agar adjusted to pH 8. We found that strain ATCC10231 failed to produce hyphae on GlcNac-plates and fewer hyphae were produced compared to strain SC5314 under the alkaline conditions present in M199 agar plates [13].

In this context we also tested the ability of the two strains to invade into a solid matrix. It is known from the literature that strain ATCC10231 is non-invasive in an intraperitoneal infection model *in vivo *[32] and our own investigations showed that this particular strain is also non-invasive in an *ex vivo *infection model [33] as well as in an *in vitro *invasion model using extracellular matrix [13]. Here we show that strain ATCC10231 is also non-invasive when incubated on agar plates depleted for carbon (YP-agar; Fig. 2B). These results indicate that while strain ATCC10231 has the capacity to produce hyphae, it is unable to do so under certain conditions, which may affect the invasive potential of this strain.

One possible attribute that has been proposed to assist invasion is the production of extracellular proteolytic and lipolytic activity. Therefore, we also tested whether strain ATCC10231 can hydrolyse extracellular proteins or lipids *in vitro*. We found that strain ATCC10231 has protease – as well as lipase/phospholipase-activity similar to strain SC5314 when tested on BSA-agar [51] and egg yolk-agar [52], respectively ([53] and data not shown).

### Strain ATCC10231 shows growth defects at alkaline pH

*In vivo *transcriptional profiling of strain SC5314 and ATCC10231 showed that *DFG16*, a gene needed for sensing external pH and response to alkaline pH values, is expressed at a lower level in strain ATCC10231 when compared with strain SC5314 during invasion into the liver [13]. Therefore, we tested the ability of strain ATCC10231 to grow on several media supplemented with compounds that may influence growth at either acidic or alkaline pH values. As shown in Fig. 2C, strain ATCC10231 showed severe growth defects when cultured on medium depleted for iron (treated with the iron chelator bathophenanthroline disulphonate (BPS)), on medium with an elevated cation concentration and on medium depleted for inorganic phosphate at pH 8. No reduction in growth was detectable when the pH of the media was adjusted to pH 5 (Fig. 2C). These observations were similar to those made with a mutant lacking the pH-sensor Dfg16 and therefore suggest that strain ATCC10231 may be unable to sense the environmental pH correctly.

### Strain ATCC10231 adheres to host cells, damages epithelial cells and survives in whole blood in a manner similar to SC5314, but has reduced potential to invade epithelial tissue

**Figure 3:**
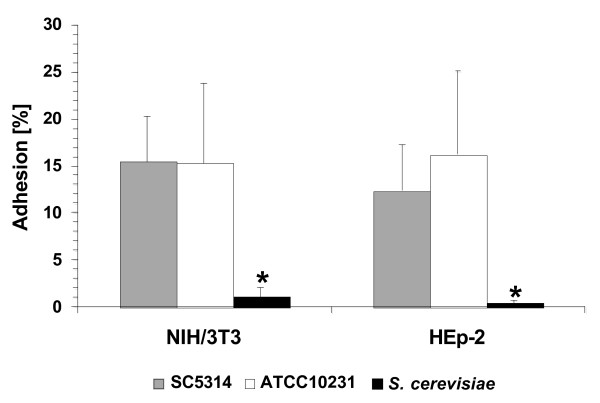
**Adhesion of *C. albicans *strains SC5314 and ATCC10231 to fibroblasts (NIH/3T3) and epithelial cells (HEp-2).** The non-adherent *S. cerevisiae *strain served as a negative control. * = significantly reduced adhesion compared to SC5314 and ATCC10231 with *P *< 0.05. Error bars = SD of n = 3 experiments.

**Figure 4:**
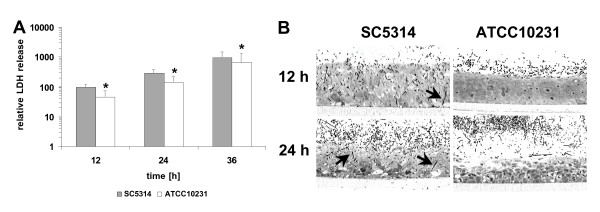
**(A) Relative LDH activity in the maintenance medium of the RHE after infection with *C. albicans *strains SC5314 and ATCC10231.** * = significantly reduced LDH-activity compared to strain SC5314 with *P *< 0.05. Error bars = SD of n = 4 experiments **(B) **Histological sections of infected RHE samples. Arrows indicate invading hyphae of strain SC5314.

In addition to biological properties that influence growth rate, the ability to attach to and invade host cells and to survive phagocytosis by immune cells are crucial attributes of *C. albicans *required to establish colonisation and to cause disease. Therefore, we analysed the interaction of strain SC5314 and ATCC10231 with different cell lines, epithelial tissue and human blood. As shown in Fig. 3, both *C. albicans *strains exhibited similar levels of adherence to epithelial cells (HEp-2) and fibroblasts (NIH/3T3) whereas very few cells of a *S*. *cerevisisae *control strain adhered to either cell line. Next, we tested the ability of the two strains to damage and to invade into a three dimensional oral tissue model based on reconstituted human oral epithelium (RHE; [54]). Although strain ATCC10231 was able to damage the surface of the tissue which is indicated by an increasing lactate dehydrogenase (LDH) activity in the maintenance medium (Fig. 4A), damage was significantly reduced and no invading hyphae could be observed in deeper tissue layers in several histological sections at time point 12 h (Fig. 4B). At later time points (24 h) strain ATCC10231 had invaded deeper tissue and had caused severe damage. This is in clear contrast to strain SC5314 which invaded into deeper cell layers of the tissue at time points 12 h and 24 h (Fig. 4B). These data indicate that strain ATCC10231 has normal adhesion properties but has reduced potential to invade host tissue. When both strains were incubated in human blood [48,49] they exhibited similar survival rates (data not shown).

### Comparative genome hybridisation (CGH) indicates no substantial genetic differences between SC5314 and ATCC10231 within the ORFs

To elucidate whether a lack of certain genes in strain ATCC10231 may explain the different biological properties identified in this study, we used a comparative genome hybridisation (CGH) protocol that has recently been applied to compare the two closely related species *C. albicans *and *C. dubliniensis*. As described by Moran *et al.*, total gDNA of both strains was enzymatically digested to produce DNA-fragments 200–2,000 basepairs in length [40]. These DNA fragments were labelled and hybridised to whole genome microarrays generated using the genome sequence of strain SC5314. After scanning we searched for differentially hybridised spots and proposed that a lack of hybridisation with the DNA from ATCC10231 would reflect the lack of a gene in this strain, while a weak hybridisation would indicate sequence differences or a higher gene copy number in SC5314.

**Figure 5:**
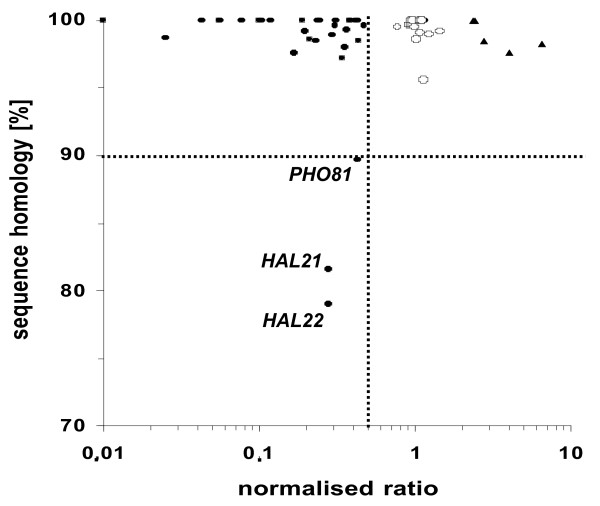
**Plot of the signal ratio and the sequence homology (compared to strain SC5314) of different genes from strain ATCC10231.** Thirty-seven genes gave at least a 2-fold weaker signal with DNA from strain ATCC10231 compared to SC5314 (filled circles). Additionally, 15 genes were included which were already sequenced and published (open circles). Filled triangles indicate 5 genes that gave a stronger signal with DNA from ATCC10231 compared to SC5314. The cut-off value for substantial differences within the sequence homology of the two strains was set at 90%. Only three genes (*PHO81*, *HAL21*, *HAL22*) fulfilled the criteria of both low signal ratio and low sequence homology.

Each spot on the microarray, representing an open reading frame (ORF) of strain SC5314, hybridised with DNA from strain ATCC10231 providing evidence that all genes identified and annotated in the genome of SC5314 also exist in strain ATCC10231. Therefore, the observed phenotypic differences cannot be explained by the lack of certain genes. However, further analysis of the CGH results identified 37 genes that gave a ≥2-fold weaker signal with DNA from strain ATCC10231 compared to DNA from strain SC5314 (see additional file 2). We also identified five genes that gave a ≥2-fold stronger signal with DNA from ATCC10231 (see additional file 2). In order to determine if these differences in hybridisation efficiency were due to divergence of the homologous nucleotide sequences in ATCC10231, fragments of these genes corresponding to the microarray target sequence were PCR-amplified from strain ATCC10231 (~300 bp) and sequenced. Additionally, we compared the sequences of 15 genes from strain ATCC10231 that were previously sequenced and published in GenBank to act as controls. Analysis of these 15 published sequences revealed that they shared ≥95% nucleotide sequence homology and exhibited similar hybridisation efficiencies (≤2-fold difference) between both strains (Fig. 5 and additional file 2). However, amplification and sequencing of the 37 gene fragments exhibiting weaker hybridisation signals from ATCC10231 DNA revealed very few nucleotide differences between strains SC5314 and ATCC10231 (Fig. 5). Only three gene fragments (from genes *PHO81*, *HAL21*, *HAL22*) were identified which showed a low signal ratio and which also had substantially different sequences (<90% homology) in the two strains SC5314 and ATCC10231 (Fig. 5). It should be noted that 11 of the analysed genes encode putative proteins encoded by retrotransposons, including the active retrotransposon Tca2 [55]. This finding suggests that the reason for the different hybidisation signals exhibited by genomic DNA from both strains may be due to copy number variations. Therefore, it can be concluded that gene sequences between strain ATCC10231 and SC5314 are highly similar or even identical throughout the entire genome.

### The transcriptional profile of strain ATCC10231 is significantly different to the profile from strain SC5314 during *in vitro *growth

*In vivo *and *ex vivo *transcriptional profiling of strains ATCC10231 and SC5314 indicated clear differences in gene expression [13]. To elucidate whether these differences were due to different responses to the host environment or whether gene expression of these strains differs even within a basic *in vitro *growth environment, we analysed the transcriptional profiles of both strains in SD-medium (pH 4.5) incubated at 37°C. For both strains, samples were collected at the late lag-phase (OD_600 _= 0.15), as this was the phase where growth of strain ATCC10231 was slightly delayed (Fig. 1). Analysis of the gene expression profiles of both strains showed that 79 genes were significantly more highly expressed in strain ATCC10231 compared to strain SC5314. No genes were detected which had significantly lower expression levels in strain ATCC10231 compared with strain SC5314. Those genes that were more highly expressed could be divided into seven major subgroups (Table 2 and additional file 3). The largest group comprised of genes with no known function (24) followed by stress-associated genes (11), and two groups of genes involved in nitrogen metabolism (8 genes). The group of nucleus- and cell surface-associated genes had seven members each. A minor group contained genes encoding proteins involved in carbohydrate metabolism (6 genes). For 16 other genes no specific groups with more than two members could be assigned.

**Table 2:** Functional categories of genes expressed at least 2-fold higher in *C. albicans *strain ATCC10231 compared with strain SC5314 after transcriptional profiling in SD-medium with *P *< 0.05

**Functional category**	**Gene names**
(oxidative) Stress	*MLS1*, *TTR1*, *GPH1*, *STI1*, *CAT1*, *SOD2*, *GLR1*, *CNB1*, *MCR1*, *YHB1, AHP1*
Nitrogen metabolism	*MEP2*, *GAT1*, *TFS1*, *orf19.5784*, *AMO2*, *HBR2*, *orf19*.*346*, *CPY1*
Nucleus associated	*HHT1*, *HTA1*, *HHF22*, *HHT21*, *NHP6A*, *orf19.4657*, *POL30*
Cell surface	*ECM33*, *ALS4*, *ALS12*, *PIR1*, *KRE1*, *PGA14*, *PGA56*
Carbon metabolism	*SFC1*, *GLK1*, *GRE3*, *SDH4*, *PMM1*, *GPM1*
Others	*ARG83*, *MNN22*, *AHP2*, *STP3*, *RIB5*, *orf19.5517*, *orf19.2166*, *orf19.5684*, *COF1*, *MIG1*, *FGR14*, *YKE2*, *ADH1*, *RIB3*, *HSL1*, *orf19.2966*
Unknown function	*orf19.5141, orf19.4132, orf19.94, orf19.1344, orf19.5612, orf19.3793, orf19.137, orf19.2693, orf19.1946, orf19.4220, orf19.868, orf19.3053, orf19.5642, orf19.3226, orf19.997, orf19.3484, orf19.4633, orf19.285, orf19.6065, orf19.949, orf19.2269, orf19.4241, orf19.6132, orf19.3335*

### Delayed growth of strain ATCC10231 in minimal medium is not due to reduced ability in the up-take of nitrogen

Within the group of genes encoding proteins involved in nitrogen metabolism we identified *MEP2*, a gene which is normally expressed under low ammonium concentrations [56]. As ammonium is the sole nitrogen source in SD-medium, we questioned whether the later entrance of strain ATCC10231 into the log-phase of growth may be connected to an altered nitrogen supply of this strain compared to strain SC5314. To test this, we analysed the ability of both strains for uptake of nitrogen sources. No significant differences were detected in ammonium uptake between these two strains. Both strains had the same capacity for the uptake of ammonium, reaching a maximum after 3 h of incubation. Additionally, changing the nitrogen source from ammonium to glutamine, glutamate, urea, or 20 different amino acids did not rescue the phenotype of delayed growth (data not shown).

## Discussion

It has long been known that different *C. albicans *strains can exhibit varying levels of virulence. For example, in one recent study, Bartie *et al. *reported the different invasive properties of *C. albicans *strains in an *in vitro *model of oral candidosis [57]. We propose that by identifying the molecular reasons for the differential virulence of candidal strains we will improve our understanding of fungal pathogenicity. Two previous studies have investigated the invasive properties of two well characterised reference strains, SC5314 and ATCC10231 *in vivo *and found that SC5314 was invasive while ATCC10231 was not [32,34]. In this study, we have used phenotypic screens, transcriptional profiling and comparative genomics to identify the principal biological and genetic properties that may determine the basis of the differential invasiveness and virulence of strain SC5314 and strain ATCC10231.

### Few distinct phenotypic differences between strains SC5314 and ATCC10231 *in vitro*

Several previous studies showed that strain SC5314 is virulent and invasive whereas strain ATCC10231 is weakly virulent and non-invasive in all infection models tested so far [27,28,32-35]. Despite this fact, we identified few *in vitro *phenotypic differences between strain SC5314 and strain ATCC10231 in this study. One of the most prominent phenotypes of strain ATCC10231 was the increased resistance of this strain to substances affecting cell membrane integrity such as itraconazole and ethanol. One explanation for this increased resistance might be an increased expression of genes from the ergosterol biosynthesis pathway. For example, it has been shown that an increased expression of *ERG11 *leads to an increased resistance towards azoles [58]. In our own previous study we noticed that strain ATCC10231 had an elevated expression of genes associated with the ergosterol biosynthesis pathway during intraperitoneal infection of mice [13] and this might also be the case for *in vitro *growth of strain ATCC10231 under the conditions tested. Elevated expression of genes from the ergosterol biosynthesis pathway would lead to an elevated ergosterol content within the plasma membrane that may contribute to an increased resistance towards azoles or other cell membrane pertubating substances. Homozygosity at the *MTL*-locus which might have an influence on azole resistance [59] can be excluded as both strains are heterozygous at the *MTL*-locus (see below).

A second prominent phenotype of strain ATCC10231 was the resistance towards 5-fluoroorotic acid (5-FOA). Usually, only strains without an intact *URA3 *gene are able to grow in the presence of 5-FOA and uracil or uridine. However, it has also been shown that chromosomal rearrangements can cause resistance towards 5-FOA although *URA3 *is still intact [60]. As the karyotype of strain ATCC10231 appears different from strain SC5314 (unpublished results B.B. Magee), chromosomal rearrangements might be one possible explanation for the resistance of strain ATCC10231 against 5-FOA.

An altered ability to form hyphae under certain circumstances was another significant phenotypic difference identified between strain SC5314 and strain ATCC10231. Strain ATCC10231 produced no hyphae in response to GlcNac and very few hyphae in M199 (pH 8) and was unable to invade into a solid matrix (YP-agar). In contrast, true hyphae of similar length were formed by both strains in an *in vitro *model of oral candidosis (RHE) and both strains were able to cause epithelial tissue damage. However, we observed that strain ATCC10231 had a significantly reduced ability to damage RHE and that the strain did not reach deeper tissue layers 12 h after inoculation, suggesting that this strain has also reduced invasive properties in the RHE model.

Reduced invasiveness of strain ATCC10231 was not associated with reduced adherence or reduced hydrolytic enzyme activity *in vitro*, since hydrolases were produced by both strains and the adhesion properties of SC5314 and ATCC10231 to different cell lines was similar. In our own previous study we showed that strain ATCC10231 had high transcript levels of genes encoding hydrolytic enzymes (such as *SAP4-6*) and genes encoding adhesion molecules (such as *ALS3 *or *HWP1*) *in vivo *[13]. Therefore, genes associated with adhesion, tissue invasion and escape from phagocytes were expressed by strain ATCC10231 during infection. The abililty of strain ATCC10231 to express these virulence factors may enable it to establish intraperitoneal infections in mice, however the inability of this strain to form hyphae under specific conditions may explain why these infections do not result in the invasion of organ tissue [32]. Furthermore, it has been described that strain SC5314 produces farnesol as a quorum sensing molecule [61] whereas strain ATCC10231 produces farnesoic acid instead [62]. Evidence suggests that farnesol may have a major influence on the outcome of infection [63], however, the exact mode of action of farnesol and farnesoic acid during infection is not known.

Clearly, invasion of *C. albicans *into host tissues is a multifactorial process [64,65] which needs to be regulated appropriately for a given environmental condition. Sensing the environmental conditions, such as the extracellular pH is therefore crucial. In this context it is of interest to note that hyphal formation of strain ATCC10231 was significantly reduced under alkaline conditions.

### Growth defects of strain ATCC10231 at alkaline pH

In a previous study we showed that strain ATCC10231 exhibits decreased expression levels of the gene *DFG16 *encoding a putative pH-sensor during intraperitoneal infection of mice [13]. Mutants of strain SC5314 lacking *DFG16 *had severe growth defects under alkaline conditions [13,18]. Therefore, we tested the ability of strain ATCC10231 to grow under several alkaline conditions. Similar to the mutant lacking *DFG16*, strain ATCC10231 showed comparable phenotypes including reduced growth in media with low content of iron or limited phosphate sources or high cation concentration at elevated pH values. These data suggest that strain ATCC10231 is not able to sense the extracellular pH correctly. Such inappropriate sensing of the extracellular pH may also explain the reduced ability of strain ATCC10231 to invade tissue.

### Phenotypic differences do not correlate with the genotypes of the two strains

It is known that the genomes of different *C. albicans *strains are highly variable [66] and to date few correlations between the phenotype and the genotype of different *C. albicans *strains have been identified. For example, it has been suggested that loss of heterozygosity at chromosome 5 and the resulting homozygosity at the *MTL*-locus can have an influence on the sensitivity to azoles [59,67] and that the mating type can also have an influence on the virulence of different *C. albicans *strains [68]. Since it is known that both strains, SC5314 and ATCC10231, are heterozygous at the *MTL*-locus [23], the observed increased resistance of strain ATCC10231 towards itraconazole cannot be explained by homozygosity at the *MTL*-locus.

Invasiveness is another phenotype that has been associated with a genetic marker in previous studies. For example, it has been proposed that the length of the 25S rDNA intron correlates with the invasive properties of different *C. albicans *strains [69]. However, since both strains investigated in this study belong to genotype A [23] our data rather support the conclusions made by Luu *et al. *who found no correlation between invasiveness and the genotype of *C. albicans *strains [70].

Comparative genome hybridisation (CGH) of strain SC5314 and strain ATCC10231 confirmed these results. CGH has, for example, previously been used for the analysis of the genomes of pathogenic and non-pathogenic bacteria [71] and the comparison of the genomes of *C. albicans *and *C. dubliniensis *[40]. By hybridising genomic DNA from strain ATCC10231 to microarrays based on the genome sequence of strain SC5314, we showed that strain ATCC10231 possesses all of the genes that are present in the genome of strain SC5314. This is in contrast to CGH studies that have compared the genomes of different *S. cerevisiae *laboratory strains [72]. In *S. cerevisiae*, genes were shown to exist in one strain but not in another [72]. In this study only 37 genes were identified that had different signal intensities after hybridisation with genomic DNA from strain ATCC10231. Of these, only three genes were found to have substantial sequence variation between the two strains. Different signal intensities may be explained by technical reasons (within the expected statistical variations when hybridizing more than 6000 genes) or by differences in ploidy or copy number of the gene between the two strains. In fact, aneuploidy seems to be widely distributed among laboratory strains of *C. albicans *[73]. Copy number differences may also explain why many genes associated with retrotransposon elements were identified in the CGH study (11 of 37 genes), including the Tca2 retrotransposon. Tca2 has been shown to be an active retrotransposon in *C. albicans *with different copy numbers among different strains [55]. Theoretically, differential activity of transposons between the two strains may have lead to differences in copy number of this retrotransposon within the two genomes.

None of the described altered phenotypes observed for ATCC10231 could be correlated to the substantial sequence differences detected for the three genes *HAL21*, *HAL22*, and *PHO81*. Of these, *HAL21 *and *HAL22 *(alias names *MET222 *and *MET22*) have been shown to lie within a highly polymorphic region of the *C. albicans *genome [26].

Although the phenotypic differences could not be explained by absent genes, differences within part of genes which do not hybridise to the microarrays or within the intergenic regions which are not represented on the microarrays may contribute to the different properties of the two strains. In particular, differences in the intergenic regions might have an impact on the transcriptional profiles of the two strains (see below). In addition, we cannot exclude that point mutations, which cannot be detected with our approach, have generated non-functional pseudogenes or resulted in subtler alterations in protein function that might explain the different phenotypic properties described here. Furthermore, it cannot be excluded that strain ATCC10231 harbours additional genes which cannot be detected on the microarrays which were designed using the genomic sequence of strain SC5314. According to the concept of pathoadaptation by loss of "antivirulence" gene function [74], it is theoretically possible that genes that are no longer compatible with an invasive lifestyle of *C. albicans *are selectively inactivated either by point mutation, insertion, or deletion.

In summary, CGH of strain SC5314 and strain ATCC10231 support the view that, despite the heterogeneity within the genomes at the physical level (as shown by electrokaryograms and similar methods) [66], *C. albicans *populations are mainly of clonal origin [75] and the gene content seems to be constant and stable between different strains. However, to confirm this hypothesis, further studies using technologies such as subtractive DNA hybridisation or genome sequencing of strain ATCC10231 are needed.

### The *in vitro *transcriptional profile cannot explain the delayed growth of strain ATCC10231

We assumed that the observed phenotypic differences between strain ATCC10231 and SC5314 may be due to either (1) the lack of certain factors, (2) the modification of certain factors or (3) differential regulation of these factors (including differential gene expression due to gene duplication or loss of an allele). To explore whether the transcriptional regulation of genes is different between the two strains, we analysed the genome wide transcriptional profile of both strains during *in vitro *growth under defined and controlled conditions. Although strains ATCC10231 and SC5314 have similar growth rates in minimal media and have an almost identical complement of genes, their transcriptional profile was clearly different. This observation is in agreement with recent studies showing that evolutionary changes in microbes, e.g. from a pathogenic to a non-pathogenic variant (or vice versa), is likely to be first detectable on the level of gene regulatory networks [76,77].

The largest group of differentially expressed genes consisted of genes with no known function. It is not clear, whether these genes are up-regulated to compensate for genetic defects in strain ATCC10231 or whether their up-regulation is directly related to the observed phenotypic and pathogenic differences between the two strains. In this study, we investigated whether up-regulation of other genes with known functions may account for the described phenotypic differences. For example, *MEP2*, a gene coding for an ammonium permease, which is usually up-regulated under low nitrogen concentrations [56], was found to be up-regulated in minimal medium. This may indicate differences in nitrogen metabolism between strains ATCC10231 and SC5314. Since certain components of the nitrogen metabolic pathway have been shown to be essential for full virulence of *C. albicans *during infection [15], we investigated whether the uptake of low molecular weight molecules such as ammonium differed in the two strains. However, no significant differences in the uptake of ammonium were observed for the two strains. Providing cells of both strains with different nitrogen sources did not modify their growth rates differentially.

In addition, *DFG16*, a gene coding for a putative pH-sensor in the Rim101 signal transduction pathway, was found to be differentially expressed in strain SC5314 and strain ATCC10231 during *in vivo *growth [13], but not during growth in minimal medium. This evidence supports the view that Dfg16 is an indispensable factor for relaying the status of environmental conditions (e.g. pH) to specific signalling pathways required for hypha formation and therefore invasion.

## Conclusion

As the total gene content seems to be very stable between the genomes of these two different *C. albicans *strains, it can be concluded that the phenotypic differences observed between these strains are likely due to changes in the expression levels of certain genes and/or due to distinct differences in the function of their encoded proteins (caused by minor sequence modifications). For example, differential regulation of *DFG16*, encoding a putative pH-sensor Dfg16, is likely to have a major impact on hypha formation, iron uptake, stress response, phosphate metabolism, invasion and virulence of *C. albicans*. A full understanding of the different factors involved in determining the invasive properties of these two widely used strains would require the complete genome sequence of strain ATCC10231 to be completed, which would allow a complete comparison of the intergenic regions and protein coding sequences of these strains.

## Authors' contributions

ST carried out the screening and molecular analysis and drafted the manuscript. GPM and DJS conceived the CGH and helped to draft the manuscript. BBM participated in the CGH and subsequent analyses. MS carried out the staining of the RHE samples. BH conceived the study, and participated in its design and coordination and helped to draft the manuscript. All authors read and approved the final manuscript.

## Supplementary Material

Additional file 1 **Oligonucleotides used in this study**. List of oligonucleotides that have been used in this study. Click here for file

Additional file 2 **Analysed genes after CGH of strain SC5314 and ATCC10231.** List of analysed genes after CGH of strain SC5314 and ATCC10231. Click here for file

Additional file 3 **Transcriptional profiling of SC5314 and ATCC10231 in SD medium.** List of genes that were significantly differential expressed in strain ATCC10231 compared with strain SC5314 after transcriptional profiling in minimal medium. Click here for file
