# Supplementary material for: Phenotypic screening, transcriptional profiling, and comparative genomic analysis of an invasive and non-invasive strain of Candida albicans
Source: BMC Microbiol. 2008 Oct 24;8:187. doi: 10.1186/1471-2180-8-187 (PMC2579918; doi:10.1186/1471-2180-8-187)
Supplement: Additional file 1 — Oligonucleotides used in this study. List of oligonucleotides that have been used in this study. [file 1471-2180-8-187-S1.doc]

**Thewes *et al*. (2008): Phenotypic screening, transcriptional profiling, and comparative genomic analysis of an invasive and non-invasive strain of *Candida albicans***

Supplementary table T1: Oligonucleotides used in this study

| **Name** |  | **Sequence (5’ - 3’)** | **Gene** |
| --- | --- | --- | --- |
| **Comparative genome hybridisation (CGH):** | | | |
| CA0037-fwd | | GCTTATGAACTTGATTTGCC | *POL93* |
| CA0037-rev | | TCACCAAACAACGATTTAGC |
| CA0069-fwd | | TCAAGGCTATTGTAATTCCG | *orf19.6468* |
| CA0069-rev | | AATGATTTGACCGTAGTTGG |
| CA0074-fwd | | AGATGGTTTGGGTGAAGTCG | *IFD7* |
| CA0074-rev | | CCTTTTCGACAACATCATTT |
| CA0286-fwd | | ACCTGAAATAAAAGCTTCCC | *orf19.6079* |
| CA0286-rev | | AAGGACTTATGTCCAACACG |
| CA0405-fwd | | AAAGCTAAATTGGCTGAATG | *orf19.5547* |
| CA0405-rev | | CACCTGGTACAAGTGGTTCT |
| CA0414-fwd | | ACAACACCATATTACCTCCG | *orf19.2164* |
| CA0414-rev | | GAAATTTGTTGTTCTCTGCC |
| CA0632-fwd | | GTTAGAATCGTCAAACACGC | *RPS5* |
| CA0632-rev | | CATCCTTCTTCTTGATAGCG |
| CA0675-fwd | | CGAGTTTCTCGACTATACCG | *orf19.1180* |
| CA0675-rev | | ACCCAAACAAAGTATCCTCC |
| CA0679-fwd | | CCTGCTGCTAATAGATCTCG | deleted from CandidaDB/CGD |
| CA0679-rev | | GTTGTGATCATTTGTATGCG |
| CA0780-fwd | | AAGCTGAAAAGGATAGAGAG | *orf19.3492* |
| CA0780-rev | | TTCGTAATATCCCCTAGTGC |
| CA0812-fwd | | AGGCATTGATATTGGTATCG | *orf19.1917* |
| CA0812-rev | | GTATAGTATTCGACGCCACC |
| CA0930-fwd | | ACAAAGTCCAAAAACAGAGC | *FGR14* |
| CA0930-rev | | ACGAGTACATTTGTTCCAGC |
| CA1141-fwd | | TGGTCAACGAATATTTAATG | *orf19.4069* |
| CA1141-rev | | TTAAATTCATCGAAAGTGGG |
| CA1142-fwd | | CAATCTGCATTACAACAAGC | *orf19.4070* |
| CA1142-rev | | ATTCCAGGTACAAAACTTCG |
| CA1149-fwd | | TGTGACTATTGGAGATTTTG | *HAL21* |
| CA1149-rev | | CGAGTTACCATAATCGATGC |
| CA1153-fwd | | GCTCTCGATTTATCCTTGAA | *orf19.104* |
| CA1153-rev | | AGGCATACTCAAGAGCCTTA |
| CA1154-fwd | | TATTGGAGATTTTGCTCTGC | *HAL22* |
| CA1154-rev | | GTTCTTGTCAGTCAATGTTC |
| CA1195-fwd | | TCTTGAAGCACTTGAATGAG | deleted from CandidaDB/CGD |
| CA1195-rev | | TTTTGTGAACTGCGATTTTA |
| CA1683-fwd | | TGGATGATCGATTAGTGAGC | *orf19.4919* |
| CA1683-rev | | TTGAACCAATTTTTCTCTGG |
| CA1852-fwd | | CCTGCTGCTAATAGATCTCG | *orf19.642.1* |
| CA1852-rev | | GTTGTGATCATTTGTATGCG |  |
| CA2216-fwd | | CGTTGAACATGAGTTAGTGG | *orf19.5372* |
| CA2216-rev | | GGAATGTCAACCAATCTAGC |
| CA2217-fwd | | TTTATGTGAATGGAAATTTG | *orf19.5373* |
| CA2217-rev | | ACGAATGCTTTGAATTTAGC |
| CA2329-fwd | | AAAATCAACAAAAGAAACAG | *orf19.4498* |
| CA2329-rev | | TTGTTTGATTTCAGATTATG |
| CA2445-fwd | | AGACAAAGATCACAACTGGC | *orf19.5503* |
| CA2445-rev | | GTAGAATGGAAATAGTGGTG |
| CA2546-fwd | | TGAACATCCATATCAACCAG | *RPC31* |
| CA2546-rev | | CATTGAATTTAATTTTGATT |
| CA3258-fwd | | TGGACTGGTTACTTACCAAT | deleted from CandidaDB/CGD |
| CA3258-rev | | AACCGTCGTATGTTGACTTC |
| CA3267-fwd | | TTCCTAACAAATTTTCGACG | *RPS620a* |
| CA3267-rev | | GGTGGCTACAGATTGATACC |
| CA3268-fwd | | TTCCTAACAAATTTTCGACG | *orf19.6301* |
| CA3268-rev | | GGTGGCTACAGATTGATACC |
| CA3330-fwd | | TTGAATGTTTCTCAGATGCC | *orf19.2669* |
| CA3330-rev | | TATCAATCGGTCTATGTGGG |
| CA3331-fwd | | TTGGTATATGATCTGGGAGC | *orf19.2668.1* |
| CA3331-rev | | GTGAATATTTCGCAATGACC |
| CA3332-fwd | | CGAAAAGATCCAATATACGC | *RHD2* |
| CA3332-rev | | AGAATAATATCATCGTGCTG |
| CA3661-fwd | | TTAAGCAATTCGAGATAGGG | *orf19.6465* |
| CA3661-rev | | CTCGTCAGAATTTAACTGGG |
| CA3984-fwd | | AAAATTTGGTCCTATTGACG | *HOM2* |
| CA3984-rev | | TGACAGCCTAATTTACTGGC |
| CA4293-fwd | | GCTCCTAATTTAAAAATTCT | *orf19.6690* |
| CA4293-rev | | AGGTCCACAAGTTCTAATCG |
| CA4303-fwd | | GGGGACAGATTATGACTTTG | *orf19.6703* |
| CA4303-rev | | CTAGATGTTTCAACTTGGAA |
| CA4390-fwd | | CATCAGGTCTCACTGAAGAA | *orf19.3122.2* |
| CA4390-rev | | TGGATTGGTTTGATTGTTTT |
| CA5385-fwd | | ATCCAAATCCAAAATACACG | *FGR24* |
| CA5385-rev | | TGTGATGATACATGGAATCG |
| CA5522-fwd | | TATTGTCTTGGGGTTACTGG | *orf19.5370* |
| CA5522-rev | | ACCAGATATCAACCCATACG |
| CA5743-fwd | | ATTATCAGTCAAAAGTGGCG | *orf19.7452* |
| CA5743-rev | | GACTTGGATAGTTTAGGAGG |
| CA5805-fwd | | TTGTTTACCAGGAAAGATGG | *orf19.5463* |
| CA5805-rev | | AATCCTGAAACTCAAAATCG |
| CA5810-fwd | | GTGTTTTCCCAGTTTTTGGT | *orf19.5468.1* |
| CA5810-rev | | GACTATAATGCACCACCTCC |
| CA5821-fwd | | ATTCGCATTCTACTTCATCG | *PHO81* |
| CA5821-rev | | ACATCATCAAATCTCAACCC |
| CA5900-fwd | | ATAGGGGTGCTAATGGTTTC | *orf19.6806* |
| CA5900-rev | | GCCGTAGAGTCAAACGTAAG |
