# Supplementary material for: Phenotypic screening, transcriptional profiling, and comparative genomic analysis of an invasive and non-invasive strain of Candida albicans
Source: BMC Microbiol. 2008 Oct 24;8:187. doi: 10.1186/1471-2180-8-187 (PMC2579918; doi:10.1186/1471-2180-8-187)
Supplement: Additional file 2 — Analysed genes after CGH of strain SC5314 and ATCC10231. List of analysed genes after CGH of strain SC5314 and ATCC10231. [file 1471-2180-8-187-S2.doc]

**Thewes et al. (2008): Phenotypic screening, transcriptional profiling, and comparative genomic analysis of an invasive and non-invasive strain of *Candida albicans***

**Supplementary table T2:** List of analysed genes after CGH of strain SC5314 and ATCC10231. In addition to the 37 weak hybridising genes from strain ATCC10231 5 genes were analysed which gave an stronger signal with DNA from strain ATCC10231 compared with DNA from strain SC5314. Grey highlighted genes are fully sequenced genes from strain ATCC10231 (GenBank).

| **Gene name CandidaDB^1^** | **Gene name CGD^2^ (GenBank accession no.)** | **Function (after CandidaDB)** | **Base exchanges** | **Homology (%)^3^** | **Array-ratio^4^** |
| --- | --- | --- | --- | --- | --- |
| *HOD1* | *orf19.3122.2* | regulator of G2/M progression (because of homology) | 3/341 | 98.3 | 6.325 |
| *Zorro2b* | *FGR24* | putative Gag protein | 7/347 | 97.7 | 3.944 |
| *Zorro1a* | *FGR14* | putative reverse transcriptase | 5/376 | 98.5 | 2.698 |
| *RPS5* | *RPS5* | ribosomal protein S5.e (because of homology) | 0/316 | 100 | 2.4 |
| *RPC31* | *RPC31* | DNA-directed RNA polymerase III (because of homology) | 0/184 | 100 | 2.31 |
| *ERG11* | *ERG11*  (AB071956) | cytochrom-P450-lanosterol 14-alpha-demethylase | 2/302 | 99.2 | 1.434 |
| *PFK1* | *PFK1*  (AJ007638) | 6-phosphofructokinase, alpha-subunit | 4/383 | 99.0 | 1.227 |
| *POL21* | *orf19.2669* | Pol part of the pCal retrotransposon | 0/313 | 100 | 1.15 |
| *EFG1* | *EFG1*  (Z32687) | enhanced filamentous growth factor | 14/308 | 95.6 | 1.126 |
| *SAP1* | *SAP1*  (X56867) | secretory aspartyl protease | 0/286 | 100 | 1.104 |
| *TUB1* | *TUB1*  (U38534) | alpha-1 tubulin | 0/383 | 100 | 1.099 |
| *RBF1.3* | *RBF1*  (D85862) | RPG-box binding factor | 3/333 | 99.1 | 1.069 |
| *BET4* | *orf19.1039*  (AB021170) | alpha-subunit of the geranylgeranyl transferase type 2 | 0/310 | 100 | 1.068 |
| *CCT8* | *CCT8*  (U37371) | component of the chaperonin-containing T-complex | 5/356 | 98.6 | 1.021 |
| *AOX1* | *AOX1*  (AF031229) | alternative oxidase (because of homology) | 2/370 | 99.5 | 0.996 |
| *INO1* | *INO1*  (L22737) | *myo*-inositol-1-phosphate synthase | 0/329 | 100 | 0.968 |
| *PFK2* | *PFK2*  (AJ007637) | 6-phosphofructokinase, alpha-subunit | 1/335 | 99.7 | 0.961 |
| *ALO1* | *ALO1*  (AF031228) | D-arabinono-1,4-lactone oxidase (because of homology) | 1/305 | 99.7 | 0.938 |
| *AOX2* | *AOX2*  (AF116872) | alternative oxidase (because of homology) | 0/321 | 100 | 0.926 |
| *SSN6* | *SSN6*  (AF170083) | transcriptional repressor (because of homology) | 1/366 | 99.7 | 0.904 |
| *URA3* | *URA3*  (X14198) | orotidine-5 -monophosphate decarboxylase | 2/388 | 99.5 | 0.768 |
| *IFC2** |  | unknown function | 1/289 | 99.6 | 0.466 |
| *IPF4153* | *orf19.5463* | similar to *S. cerevisiae* Sec6 protein (because of homology) | 4/304 | 98.5 | 0.432 |
| *PHO81* | *PHO81* | cyclin-dependent kinase inhibitor (because of homology) | 27/304 | 89.7 | 0.428 |
| *IPF14618* | *orf19.6079* | unknown function | 0/327 | 100 | 0.427 |
| *IPF19862* | *orf19.5547* | unknown function | 0/115 | 100 | 0.418 |
| *IPF708* | *orf19.5370* | unknown function | 0/328 | 100 | 0.385 |
| *IPF4137* | *orf19.5468.1* | unknown function | 0/239 | 100 | 0.378 |
| *IPF4784* | *orf19.4498* | unknown function | 2/307 | 99.3 | 0.363 |
| *IPF13098* | *orf19.1180* | unknown function | 7/392 | 98.0 | 0.355 |
| *IPF2895* | *orf19.7452* | unknown function | 8/327 | 97.2 | 0.34 |
| *Cirt1a* | *orf19.3492* | transposase | 0/329 | 100 | 0.31 |
| *RPS620a* | *RPS620a* | unknown function | 1/278 | 99.6 | 0.305 |
| *IPF3348* | *orf19.1917* | unknown function | 3/310 | 98.9 | 0.293 |
| *MET223* | *HAL21* | Ser/Thr protein phosphatase (because of homology) | 37/241 | 81.6 | 0.28 |
| *MET221* | *HAL22* | Ser/Thr protein phosphatase (because of homology) | 35/202 | 79.0 | 0.277 |
| *IPF13885* | *orf19.5503* | unknown function | 0/180 | 100 | 0.245 |
| *IFD7* | *IFD7* | putative aryl-alcohol dehydrogenase (because of homology) | 0/195 | 100 | 0.238 |
| *KAR5** |  | nuclear fusion protein (because of homology) | 4/335 | 98.6 | 0.211 |
| *RPS620b* | *orf19.6301* | unknown function | 2/278 | 99.2 | 0.198 |
| *IPF2379* | *orf19.6806* | unknown function | 4/205 | 97.6 | 0.167 |
| *IPF17322* | *orf19.4069* | unknown function | 0/341 | 100 | 0.118 |
| *IFB1* | *orf19.6703* | unknown function | 0/164 | 100 | 0.104 |
| *Cirt5* | *orf19.4919* | putative transposase | 0/359 | 100 | 0.1 |
| *IPF17991* | *orf19.6465* | unknown function | 0/323 | 100 | 0.077 |
| *IFA8* | *orf19.6690* | unknown function | 0/327 | 100 | 0.056 |
| *IPF17652.3* | *POL93* | reverse transcriptase (because of homology) | 0/308 | 100 | 0.055 |
| *HOM2* | *HOM2* | aspartate-semialdehyde dehydrogenase (because of homology) | 0/366 | 100 | 0.043 |
| *IPF4068* | *orf19.2164* | reverse transcriptase | 4/348 | 98.7 | 0.025 |
| *POL21.53f** |  | Gag protein of the pCal retrotransposon | 0/327 | 100 | 0.01 |
| *IPF10455* | *orf19.104* | unknown function | 0/375 | 100 | 0.01 |
| *IPF19295* | *orf19.6468* | unknown function | 0/363 | 100 | 0.01 |
| *IPF6235* | *orf19.5372* | Tca2 retrotransposon | 0/315 | 100 | 0.01 |
| *IPF9459* | *orf19.4070* | unknown function | 0/394 | 100 | 0.01 |
| *POL0* | *orf19.5373* | Pol polyprotein, reverse transcriptase | 0/367 | 100 | 0.01 |

^1^http://genolist.pasteur.fr/CandidaDB/; ^2^http://www.candidagenome.org/; ^3^Homolgy of ATCC10231 sequence compared with SC5314 sequence; ^4^signal from ATCC10231 compared with signal from SC5314; *not found in CGD.
