# Supplementary material for: Phenotypic screening, transcriptional profiling, and comparative genomic analysis of an invasive and non-invasive strain of Candida albicans
Source: BMC Microbiol. 2008 Oct 24;8:187. doi: 10.1186/1471-2180-8-187 (PMC2579918; doi:10.1186/1471-2180-8-187)
Supplement: Additional file 3 — Transcriptional profiling of SC5314 and ATCC10231 in SD medium. List of genes that were significantly differential expressed in strain ATCC10231 compared with strain SC5314 after transcriptional profiling in minimal medium. [file 1471-2180-8-187-S3.doc]

**Thewes *et al*. (2008): Phenotypic screening, transcriptional profiling, and comparative genomic analysis of an invasive and non-invasive strain of *Candida albicans***

Supplementary table T3: Genes significantly differential expressed in strain ATCC10231 compared with strain SC5314 after transcriptional profiling in minimal medium.

| **Name^a^** | **orf19-No.^a^** | **Description** | **normalised ratio^b^** | ***t*-test**  ***P*-value^c^** |
| --- | --- | --- | --- | --- |
| *MEP2* | *orf19.5672* | high affinity low capacity ammonium permease (by homology) | 20.4 | 0.032 |
|  | *orf19.5141* | unknown function | 13.9 | 0.030 |
| *MLS1* | *orf19.4833* | malate synthase | 12.7 | 1.13E-07 |
| *AMO1* | *orf19.5784* | amine oxidase (by homology) | 11.6 | 0.044 |
| *YHB1* | *orf19.3707* | flavohemoglobine (by homology) | 10.9 | 0.002 |
| *ARG83* | *orf19.2748* | zinc finger transcription factor of the Zn(2)-Cys(6)-binuclear cluster domain type (by homology) | 6.3 | 6.38E-04 |
| *MNN22* | *orf19.11284* | golgi α-1,2-mannosyltransferase (by homology) | 5.6 | 4.23E-05 |
|  | *orf19.4132* | unknown function | 5.3 | 0.013 |
| *SFC1* | *orf19.3931* | succinate fumarate transporter (by homology) | 5.2 | 0.002 |
| *ALS4* | *orf19.4556* | agglutinin-like protein | 5.1 | 0.001 |
|  | *orf19.94* | unknown function | 5.1 | 0.002 |
|  | *orf19.1344* | unknown function | 4.6 | 4.98E-04 |
|  | *orf19.5612* | unknown function | 4.6 | 0.020 |
| *AHP2* | *orf19.6470* | peroxidase activity | 4.4 | 0.003 |
|  | *orf19.3793* | unknown function | 4.3 | 8.14E-04 |
|  | *orf19.137* | unknown function | 4.3 | 0.002 |
| *ALS12* | *orf19.2122* | agglutinin-like protein | 4.2 | 3.04E-04 |
| *GLK4* | *orf19.6116* | aldohexose specific glucokinase (by homology) | 4.1 | 8.37E-04 |
| *GAT1* | *orf19.1275* | nitrogen regulation (by homology) | 4.1 | 8.41E-04 |
|  | *orf19.2693* | unknown function | 4.0 | 4.43E-04 |
|  | *orf19.4657* | needed for nuclear morphology (by homology) | 4.0 | 0.001 |
| *STP3* | *orf19.5917* | similar to *S. cerevisiae* Stp2 (by homology) | 3.8 | 9.61E-04 |
|  | *orf19.5517* | alcohol dehydrogenase (by homology) | 3.5 | 3.40E-04 |
|  | *orf19.1946* | unknown function | 3.5 | 0.004 |
| *AMO2* | *orf19.3152* | amine oxidase (by homology) | 3.5 | 0.011 |
| *RIB5* | *orf19.4024* | riboflavine synthase (by homology) | 3.3 | 3.93E-04 |
| *TFS1* | *orf19.1974* | Cdc25-dependent cell cycle regulator of the nutrient and ammonium response (by homology) | 3.3 | 0.008 |
| *AHP1* | *orf19.2762* | alkyl hydroperoxide reductase | 3.2 | 2.87E-04 |
| *TTR1* | *orf19.6059* | glutaredoxine (by homology) | 3.1 | 6.36E-04 |
| *KRE1* | *orf19.4377* | protein of the secretory pathway | 3.0 | 0.002 |
|  | *orf19.2966* | putative carboxymethylenebutenolidase (dienlacton hydrolase, DLH) (by homology) | 3.0 | 0.003 |
| *RIB3* | *orf19.5228* | 3,4-dihydroxy-2-butanon-4-phosphate synthase (by homology) | 3.0 | 0.003 |
| *ADH1* | *orf19.3997* | alcohol dehydrogenase (by homology) | 2.9 | 3.39E-04 |
|  | *orf19.4220* | unknown function | 2.7 | 0.009 |
| *ADAEC* | *orf19.868* | Unknown function | 2.7 | 0.010 |
| *PGA14* | *orf19.968* | GPI-anchored protein | 2.6 | 7.37E-04 |
| *YKE2* | *orf19.6601.1* | Gim complex component, 3’-end (by homology) | 2.6 | 0.002 |
| *HSL1* | *orf19.4308* | Ser/Thr protein kinase (by homology) | 2.5 | 0.013 |
|  | *orf19.3053* | unknown function | 2.4 | 0.004 |
|  | *orf19.5642* | unknown function | 2.4 | 0.004 |
| *GPH1* | *orf19.7021* | glycogen phosphorylase (by homology) | 2.4 | 0.004 |
|  | *orf19.3226* | unknown function | 2.4 | 0.006 |
| *HBR2* | *orf19.1078* | similarity with different transaminases, 3’-end (by homology) | 2.4 | 0.011 |
| *GRE3* | *orf19.4317* | D-xylose reductase (by homology) | 2.4 | 0.015 |
| *FGR14* | *orf19.559* | putative reverse transcriptase | 2.4 | 0.017 |
|  | *orf19.997* | unknown function | 2.3 | 0.012 |
| *MIG1* | *orf19.4318* | transcriptional regulator | 2.3 | 0.013 |
|  | *orf19.3484* | unknown function | 2.3 | 0.024 |
| *COF1* | *orf19.953.1* | cofilin (by homology) | 2.2 | 0.003 |
| *PIR1* | *orf19.220* | putative cell wall protein of the PIR family | 2.2 | 0.009 |
|  | *orf19.4633* | unknown function | 2.2 | 0.010 |
| *SDH4* | *orf19.4022* | succinate dehydrogenase membrane anchor subunit for Sdh2 (by homology) | 2.2 | 0.010 |
|  | *orf19.285* | unknown function | 2.2 | 0.015 |
|  | *orf19.6065* | unknown function | 2.2 | 0.034 |
|  | *orf19.5684* | ribosomale protein of the large subunit (L14), mitochondrial, by homology | 2.1 | 0.005 |
| *STI1* | *orf19.10702* | stress-induced protein (by homology) | 2.1 | 0.010 |
| *CNB1* | *orf19.4009* | protein phosphatase, calcineurin B (by homology) | 2.1 | 0.013 |
|  | *orf19.949* | unknown function | 2.1 | 0.022 |
|  | *orf19.2166* | H^+^-ATPase V1 domain; 42 kDa subunit (by homology) | 2.0 | 0.025 |
| *CAT1* | *orf19.6229* | catalase A, peroxisomal (by homology) | 2.0 | 0.013 |
|  | *orf19.2269* | unknown function | 2.0 | 0.017 |
|  | *orf19.346* | putative alanin transaminase (by homology) | 2.0 | 0.018 |
| *POL30* | *orf19.4616* | proliferating nuclear antigen (by homology) | 1.9 | 0.013 |
| *HHT1* | *orf19.6791* | histon H3 | 1.9 | 0.007 |
| *HTA1* | *orf19.6924* | histon H2A (by homology) | 1.9 | 0.033 |
|  | *orf19.4241* | unknown function, 3’-end | 1.9 | 0.035 |
| *HHF22* | *orf19.1854* | histon H4 (by homology) | 1.8 | 0.008 |
| *PGA56* | *orf19.1105.2* | unknown function | 1.8 | 0.009 |
| *ECM33* | *orf19.3010.1* | cell wall biogenesis, 3’-end (by homology) | 1.8 | 0.013 |
| *HHT21* | *orf19.1061* | histon H3 | 1.8 | 0.032 |
|  | *orf19.6132* | unknown function | 1.8 | 0.037 |
| *PMM1* | *orf19.2937* | phosphomannomutase | 1.7 | 0.019 |
| *CPY1* | *orf19.8919* | carboxypeptidase Y-precursor, 3’-end | 1.7 | 0.027 |
| *GLR1* | *orf19.11623* | by homology to *S. cerevisiae* and *C. albicans;* glutathione reductase | 1.7 | 0.033 |
| *SOD2* | *orf19.3340* | manganese superoxide dismutase | 1.7 | 0.034 |
| *NHP6A* | *orf19.3335* | chromosomale protein | 1.6 | 0.039 |
|  | *orf19.3335* | unknown function | 1.6 | 0.038 |
| *GPM1* | *orf19.903* | phosphoglycerat mutase (by homology | 1.6 | 0.033 |
| *MCR1* | *orf19.3507* | NADH-cytochrome-b5 reductase (by homology) | 1.6 | 0.036 |

^a^www.candidagenome.org; ^b^ATCC10231 compared with SC5314; ^c^Student’s *t*-test
